# Effects of Kaempferol Supplementation on the Cryopreservation Quality of Semen from Yuansheng Aite Dairy Rams

**DOI:** 10.3390/antiox15060773

**Published:** 2026-06-22

**Authors:** Guoliang Wang, Jiahao Han, Sitong Jia, Siyuan Fan, Zhongshi Zhu, Shuxian Guo, Naseer Ahmad, Bin Zhang, Yuxuan Song, Lei Zhang

**Affiliations:** 1 College of Animal Science and Technology, Northwest A&F University, Yangling 712100, China; 2024055462@nwafu.edu.cn (G.W.);; 2 College of Life Sciences, Northwest A&F University, Yangling 712100, China

**Keywords:** sperm motility, antioxidant defense, ATP metabolism, dose–response, multi-omics

## Abstract

Sperm cryopreservation is important for livestock breeding and germplasm conservation, but freeze–thaw injury can impair ram sperm quality through oxidative stress, membrane damage, and metabolic disturbance. This study evaluated the concentration-dependent effects of kaempferol supplementation on the cryopreservation quality of semen from Yuansheng Aite dairy rams. Qualified ejaculates were pooled and randomly allocated to five equally spaced kaempferol treatment groups: 0, 25, 50, 75, and 100 μg/mL. Post-thaw sperm motility, oxidative stress status, ATP-related energy metabolism, acrosome integrity, and multi-omics profiles were evaluated. Data were analyzed using appropriate parametric or non-parametric tests after assessment of normality and homogeneity of variance. Orthogonal polynomial analysis was performed to evaluate linear and nonlinear dose–response patterns across the tested kaempferol concentrations. Kaempferol supplementation significantly affected PM, VCL, and VAP, while RPM, LIN, WOB, and VSL were not significantly affected. No significant linear effect was observed for the motility parameters, whereas VCL exhibited a significant quadratic response to kaempferol concentration. Based on the observed overall responses of sperm motility, antioxidant capacity, oxidative stress markers, ATP content, and acrosome integrity, 25 μg/mL kaempferol showed the most favorable overall profile among the tested concentrations and was selected for subsequent mechanistic analyses. Proteomic and metabolomic analyses suggested that the protective effects of kaempferol may be associated with pathways related to focal adhesion, cytoskeletal organization, oxidative phosphorylation-related energy metabolism, and central carbon metabolism. These findings indicate that moderate kaempferol supplementation may improve the post-thaw quality of Yuansheng Aite dairy ram semen, although further fertility-oriented studies are needed to confirm its practical reproductive benefits.

## 1. Introduction

Livestock germplasm forms the foundation of modern animal husbandry systems [1]. As a core component of biodiversity, livestock genetic resources constitute not only the material foundation for human survival and development but also vital strategic assets for sustainable agricultural development and national food security [2]. Livestock farming currently accounts for approximately 40% of total agricultural output value [3]. Within this sector, sheep genetic resources constitute a vital component of global livestock biodiversity [4,5]. Localized breeds, with their unique adaptability and productive performance, serve as core assets for establishing indigenous industrial systems [6,7]. The Yuansheng Aite dairy sheep represents China’s first entirely domestically developed dairy sheep breed with full intellectual property rights. However, the breed currently faces risks of genetic drift and inbreeding depression due to its small population size and concentrated distribution. Establishing an efficient and stable semen cryopreservation system is to achieve off-site genetic backup and cross-regional utilization.

Artificial insemination with frozen-thawed semen is an important reproductive technology for accelerating genetic improvement and expanding the use of superior dairy rams. However, the cryopreservation efficiency of ram semen, including semen from Yuansheng Aite dairy rams, remains constrained by freeze–thaw injury. Cooling, freezing, and thawing commonly reduce sperm motility, impair plasma membrane and acrosome integrity, disturb ATP-related energy metabolism, and decrease fertilizing potential [8,9,10,11,12,13,14,15]. These injuries are closely associated with excessive reactive oxygen species (ROS) production and membrane lipid peroxidation, because ram sperm membranes are rich in unsaturated fatty acids and spermatozoa possess limited cytoplasmic antioxidant defenses [14,16,17,18,19,20]. Commercial extenders developed for ram semen are widely used across breeds and can provide acceptable cryoprotection. Nevertheless, breed-related differences in sperm membrane composition, seminal plasma characteristics, antioxidant capacity, and cryotolerance may influence post-thaw semen quality. Thus, breed-specific evaluation of extender supplementation remains necessary for optimizing the cryopreservation of Yuansheng Aite dairy ram semen.

Antioxidant supplementation of semen extenders has been widely investigated as a biochemical strategy to alleviate cryopreservation-induced sperm damage [21,22,23,24,25]. Previous studies have shown that antioxidants can improve frozen-thawed semen quality by reducing oxidative stress, limiting lipid peroxidation, preserving membrane integrity, and supporting sperm energy metabolism [11,21,22,23,24,25]. Recent advances in natural biomolecules and plant-derived compounds have further positioned natural antioxidants as promising semen extender additives because of their safety profile and multi-target protective capacity [26]. However, antioxidant efficacy is not universally linear and may depend on species, extender formulation, concentration, and sperm redox physiology. Excessive antioxidant supplementation may disrupt physiological ROS-mediated signaling required for normal sperm function. Therefore, a properly arranged dose–response evaluation is required before a natural antioxidant can be recommended for semen cryopreservation.

Kaempferol, a flavonoid compound widely present in vegetables and fruits, has been demonstrated to effectively scavenge ROS, inhibit lipid peroxidation, and stabilize mitochondrial membrane potential [27,28,29,30]. Recent studies indicate that oxidative stress, mitochondrial dysfunction, membrane lipid peroxidation, and structural damage are central determinants of sperm cryoinjury, particularly in small ruminants, making antioxidant-based extender optimization a rational strategy [31]. Recent bovine studies further suggest that kaempferol supplementation can improve post-thaw sperm function, attenuate oxidative damage, and stabilize sperm membranes, whereas evidence in ram semen remains limited [32].

Therefore, we hypothesized that kaempferol supplementation would affect the post-thaw quality of semen from Yuansheng Aite dairy rams in a concentration-dependent manner. A moderate concentration of kaempferol is expected to yield superior cryoprotective effects compared with low or excessive doses. Accordingly, this study was conducted to investigate the impacts of graded kaempferol concentrations on sperm motility, antioxidant capacity, oxidative stress, energy metabolism, acrosome integrity and omics profiles of frozen-thawed semen, and to identify the most effective tested concentration for semen cryopreservation under the present experimental conditions.

## 2. Materials and Methods

### 2.1. Ethical Statement

The YuanSheng AiTe dairy rams employed in this study were sourced from Gansu Yuansheng Agriculture & Animal Husbandry Technology Co., Ltd. (Jinchang, China). All animal procedures received approval from the Institutional Animal Care and Use Committee of Northwest A&F University (Approval No. IACUC2024-1210, approved on 9 November 2024). Animal experiment reporting was guided by the ARRIVE 2.0 guidelines [33].

### 2.2. Preparation of Semen Cryopreservation Diluents

Kaempferol (purity ≥ 97%) was purchased from Macklin Biochemical Co., Ltd. (Shanghai, China; Cat. No. K812226; CAS No. 520-18-3) via its authorized distributor, Shandong Keyuan Biochemical Co., Ltd. (Laizhou, China). Kaempferol was first dissolved in dimethyl sulfoxide (DMSO) to prepare a 10 mg/mL concentrated stock solution. Cryopreservation Diluent Solution I was prepared prior to semen collection: 2.7 g of Tris, 1.0 g of glucose, and 1.4 g of citric acid were accurately weighed and dissolved in 80 mL of ultrapure water, followed by pH adjustment to 7.0. The prepared solution was filtered through a 0.22 μm sterile filter membrane and then supplemented with 50,000 units of penicillin, 50,000 units of streptomycin, and 20 mL of egg yolk. Immediately before semen dilution, five independent extender aliquots were prepared separately for the 0, 25, 50, 75, and 100 μg/mL kaempferol treatment groups. Corresponding volumes of the kaempferol stock solution were added separately to each treatment extender to obtain the designated final kaempferol concentrations. To exclude the potential interference of DMSO, the final concentration of DMSO was maintained at 1.0% (*v*/*v*) across all groups. Specifically, 0, 2.5, 5.0, 7.5, and 10.0 μL of kaempferol stock solution were added per millilitre of final diluted semen for the 0, 25, 50, 75, and 100 μg/mL groups, respectively, while 10.0, 7.5, 5.0, 2.5, and 0 μL of pure DMSO were supplemented accordingly to balance the total DMSO volume. The 0 μg/mL kaempferol group served as the vehicle control. All treatment extenders were fully homogenized and maintained at 38 °C before semen dilution. The Tris-citric acid-glucose-egg yolk extender and freezing extender preparation were performed with reference to published ram semen cryopreservation protocols, with minor modifications [34,35].

Cryopreservation Diluent Solution II was prepared immediately prior to semen dilution: 6% (*v*/*v*) glycerol was added to the pre-prepared Solution I, and the pH of the mixture was readjusted to 7.0. The resulting Solution II was transferred to a 4 °C refrigerator and stored until use.

### 2.3. Semen Collection and Processing

Six healthy, sexually mature Yuansheng Aite dairy rams were used for semen collection. Semen was collected using the artificial vagina method. Immediately after collection, ejaculates were maintained at 37 °C and transported to the laboratory within 30 min. Fresh semen quality was evaluated microscopically at 37 °C, and only ejaculates meeting the following criteria were included: no foreign matter or abnormal odour, milky white or creamy yellow colour, sperm abnormality rate < 5%, total motility ≥ 80%, sperm concentration ≥ 1.5 × 10^9^/mL, and typical wave-like motility under microscopy.

For each independent experimental replicate, eligible ejaculates collected at the same sampling time were pooled at 37 °C to form one replicate-specific semen master batch. This pooling strategy was used to reduce the influence of individual ram variation during treatment comparison. Each master batch was then divided into equal aliquots and assigned to the corresponding treatment groups. Semen collection, initial quality assessment, and pooling procedures were performed according to published ram semen cryopreservation protocols, with minor modifications [34,35].

### 2.4. Experimental Design and Cryopreservation

A completely randomized design was used in this study. For the concentration-screening experiment, each independently prepared pooled semen master batch was divided into five equal aliquots, and the aliquots were randomly assigned to five kaempferol treatment groups: 0, 25, 50, 75, and 100 μg/mL. Thus, within each independent replicate, one aliquot was prepared for each kaempferol concentration. The experimental unit was the pooled semen aliquot assigned to a given kaempferol treatment within each independent replicate. For sperm motility analysis, three independent replicates were included for each treatment concentration.

Based on the concentration-screening results, the control group and the 25 μg/mL kaempferol-treated group were selected for subsequent oxidative stress, energy metabolism, acrosome integrity, proteomic, and metabolomic analyses. For the proteomic and metabolomic analyses, six independent biological replicates were included in each group. Specifically, six independently prepared freezing batches were generated. In each batch, qualified ejaculates were pooled to form one master batch, which was then divided into paired aliquots assigned to the control group and the 25 μg/mL kaempferol-treated group, producing paired samples CK-1/TG-1 through CK-6/TG-6. Therefore, these samples represented independent biological replicates rather than repeated technical injections of the same sample.

The diluted semen was loaded into 0.25-mL straws, sealed, and equilibrated at 4 °C for 2 h. After equilibration, the straws were placed horizontally 3 cm above the surface of liquid nitrogen for 15 min and then plunged into liquid nitrogen for storage. For post-thaw analysis, straws were thawed in a 37 °C water bath for 30 s before evaluation. The cryopreservation procedure was adapted from published ram semen freezing protocols [34,35].

### 2.5. Computer-Assisted Sperm Analysis (CASA)

Computer-Assisted Semen Analysis (CASA, Beijing Saise Medical Technology Co., Ltd., Beijing, China) was employed to assess sperm motility and kinematic parameters in both fresh and thawed semen samples. Sperm motility and kinematic parameters were assessed using CASA according to published methods for ram sperm motility evaluation [36].

Analysis parameters for fresh semen included total sperm motility (PM, %), progressive motility (RPM, %), and the following kinematic parameters: straight-line velocity (VSL, µm/s), curved-line velocity (VCL, µm/s), average path velocity (VAP, µm/s), linearity (LIN, %), and wobble (WOB, %).

For thawed semen samples, the cryopreservation straws were first placed in a 37 °C water bath for 30 s to thaw, then analysed using the same parameters as above.

### 2.6. Oxidative Stress and Energy Metabolism Marker Assays

The assays were performed using frozen-thawed semen suspensions rather than isolated sperm extracts. After thawing, samples were mixed thoroughly to ensure homogeneity and aliquoted for each biochemical assay. According to the manufacturers’ specifications, these kits are suitable for animal tissue homogenates, cell lysates, and biological fluid samples, and were applied here to frozen-thawed semen samples. Oxidative stress and energy metabolism indicators were measured according to the manufacturers’ instructions and published methods for biochemical assessment of cryopreserved ram sperm, with minor modifications [35,37]. The detailed information of detected indicators and corresponding kits is as follows: Total antioxidant capacity (T-AOC): Total Antioxidant Capacity Assay Kit, Beijing Box Biotech Co., Ltd. (Beijing, China), Cat. No.: AKAO012C. Malondialdehyde (MDA): Malondialdehyde Assay Kit, Beijing Box Biotech Co., Ltd. (Beijing, China), Cat. No.: AKFA013M. Superoxide dismutase (SOD): Superoxide Dismutase Activity Assay Kit (WST-8 method), Beijing Box Biotech Co., Ltd. (Beijing, China). Catalase (CAT): Catalase Activity Assay Kit (visible colourimetric method), Beijing Box Biotech Co., Ltd. (Beijing, China). NADPH oxidase (NOX): NADPH Oxidase Assay Kit, Beijing Box Biotech Co., Ltd. (Beijing, China). Reactive oxygen species (ROS): Reactive Oxygen Species Assay Kit (chemiluminescence method), Beijing Box Biotech Co., Ltd. (Beijing, China). Adenosine triphosphate (ATP): Adenosine Triphosphate Content Assay Kit (phosphomolybdic acid colorimetric method), Beijing Box Biotech Co., Ltd. (Beijing, China).

### 2.7. Metabolomics Sequencing and Analysis

For metabolomic analysis, frozen-thawed semen samples from the control group and the 25 μg/mL kaempferol-treated group were used, with six independent biological replicates included in each group. Each replicate was derived from an independently prepared freezing batch, as described in Section 2.4.

During the analysis, one quality control sample was inserted every 5–10 experimental samples to perform untargeted metabolomic profiling on frozen-thawed ram semen samples. The analysis was conducted on a Thermo Fisher UHPLC-Orbitrap Exploris 240 system (Thermo Fisher Scientific, Waltham, MA, USA) equipped with an HSS T3 column (100 mm × 2.1 mm i.d., 1.8 μm particle size; Waters Corporation, Milford, MA, USA). The mobile phase comprised 0.1% formic acid in water-acetonitrile (95:5, *v*/*v*) as Phase A and 0.1% formic acid in acetonitrile-isopropanol-water (47.5:47.5:5, *v*/*v*/*v*) as Phase B, with a flow rate of 0.40 mL/min and the column temperature maintained at 40 °C. Mass spectrometry data were acquired in both positive and negative ion modes with a scan range of *m*/*z* 70–1050. The key MS parameters were as follows: spray voltage of 3500 V (positive mode) and −3000 V (negative mode), sheath gas flow rate of 50 arbitrary units, auxiliary gas flow rate of 13 arbitrary units, ion source temperature of 450 °C, and stepped collision energy of 20–40–60 V. Raw LC-MS data were processed by Progenesis QI software (v3.0, Waters Corporation, Milford, MA, USA) for baseline correction, peak identification and retention time alignment, to generate a data matrix including retention time, *m*/*z* and peak intensity. Metabolite identification was achieved by matching MS and MS/MS spectra with HMDB, Metlin databases and in-house library. Data preprocessing included missing value filtering according to the 80% rule, sum normalization, removal of variables with relative standard deviation > 30% in QC samples and log10 transformation. Principal component analysis and orthogonal partial least squares discriminant analysis were performed using the ropls package in R language (v4.6.0; R Foundation for Statistical Computing, Vienna, Austria), with model stability evaluated by 7-fold cross-validation. Differentially expressed metabolites (DEMs) were screened based on variable importance in projection (VIP) > 1 and *p* < 0.05, and functional annotation and topological analysis of DEMs were conducted via the KEGG database. Data analysis employed PCA, PLS-DA, and OPLS-DA dimensionality reduction using the R Ropls package (v1.22.0). Replacement tests validated models against overfitting. Differential metabolite screening criteria: *p*-value < 0.05 and VIP > 1, supplemented by fold change (FC) analysis. Differentially expressed metabolites underwent hypergeometric distribution testing and intermediary centrality topological analysis for KEGG pathway enrichment, visualized using KEGG Mapper (Kanehisa Laboratories, Kyoto, Japan) alongside R/pheatmap packages. Untargeted metabolomic profiling, multivariate statistical analysis, metabolite annotation, and pathway enrichment were performed according to published LC-MS-based metabolomics workflows and database resources, including ropls, HMDB, METLIN, and KEGG [38,39,40,41].

### 2.8. Proteomics Sequencing and Analysis

For proteomic analysis, frozen-thawed semen samples from the control group and the 25 μg/mL kaempferol-treated group were used, with six independent biological replicates included in each group. Each replicate was derived from an independently prepared freezing batch, as described in Section 2.4.

Frozen-thawed semen samples were lysed in lysis buffer containing 8 M urea, 1 mM PMSF, and 2 mM EDTA, followed by sonication in an ice bath for 5 min. After centrifugation at 15,000× *g* for 10 min at 4 °C, the supernatant was collected for protein quantification using a BCA assay. An aliquot of 100 μg protein was reduced with 5 mM DTT at 37 °C for 45 min, alkylated with 11 mM iodoacetamide in the dark for 15 min, and digested with trypsin overnight at 37 °C. The resulting peptides were desalted using C18 columns (Millipore, Billerica, MA, USA) and quantified with a peptide quantification kit (Thermo Fisher Scientific, Waltham, MA, USA). Peptide separation was performed on a Thermo Fisher Vanquish Neo UHPLC nano-liquid chromatography system (Thermo Fisher Scientific, Waltham, MA, USA). Mobile phase A consisted of 0.1% formic acid in water, and mobile phase B consisted of 0.1% formic acid in acetonitrile. The separation was conducted at 55 °C with an injection amount of 200 ng and a flow rate of 2.5 μL/min. Mass spectrometry was carried out in positive ion mode using a Thermo Fisher Orbitrap™ Astral™ high-resolution mass spectrometer (Thermo Fisher Scientific, Waltham, MA, USA) in DIA mode. Raw data were processed with DIA-NN software (v1.8.1) against the sheep proteome database (UP000002356), with the false discovery rate (FDR) set to 1% at both precursor and protein levels. Protein quantification was performed using the MaxLFQ algorithm. Differentially expressed proteins were identified using thresholds of fold change ≥ 1.5 or ≤0.6667 and *p* ≤ 0.05, followed by GO, KEGG, KOG annotation and domain enrichment analysis. Proteomic sample preparation, DIA-MS data processing, and label-free protein quantification were performed according to published proteomic workflows and software methods, with minor modifications [42,43].

### 2.9. Sperm Acrosome Integrity Assessment

Sperm acrosome integrity was assessed using FITC-PNA/DAPI dual-fluorescent staining: Take 10 μL of semen sample and mix with equal volumes of FITC-PNA staining solution (final concentration 10 μg/mL) and DAPI staining solution (final concentration 1 μg/mL). Incubate in the dark at 37 °C for 15 min before preparing slides. Observe under a fluorescence microscope (excitation wavelengths 488 nm and 350 nm respectively). Randomly count no fewer than 200 spermatozoa. Those simultaneously displaying green (FITC-PNA) and blue (DAPI) fluorescence are classified as having intact acrosomes. The acrosome integrity rate is calculated as the percentage of acrosomal intact spermatozoa relative to the total counted spermatozoa. Each sample was observed in 3 random fields under a fluorescence microscope, no less than 200 spermatozoa were counted in total for each technical replicate, and the acrosome integrity rate was calculated for each technical replicate. Acrosome integrity was evaluated using FITC-PNA/DAPI staining according to published sperm acrosome assessment methods, with minor modifications [37,44].

### 2.10. Bioinformatics Integrated Analysis

First, KEGG enrichment analysis (KEGG Mapper, http://www.kegg.jp/kegg/mapper.html, accessed 14 May 2025) to identify overlapping metabolic pathways. Subsequently, Pearson correlation analyses were performed between DEPs/DEMs enriched in these overlapping pathways and post-thaw sperm motility to calculate correlation coefficients. Furthermore, candidate biomarkers for sperm cryopreservation were prioritized based on their centrality within enriched pathways, experimental support from cryopreservation-related literature, and significant correlation with post-thaw sperm motility (absolute correlation coefficient |r| ≥ 0.6 and *p* < 0.05). Bioinformatics integration was conducted using KEGG pathway enrichment and correlation-based association analysis according to published omics analysis strategies [38,41].

### 2.11. Statistical Analysis

Data are presented as mean ± standard deviation (SD). The number of independent replicates used for each assay is indicated in the corresponding table or figure legend. For sperm motility analysis in the concentration-screening experiment, three independent replicates were included for each kaempferol concentration. For proteomic and metabolomic analyses, six independent biological replicates were included in each group.

Before statistical analysis, data were assessed for normality and homogeneity of variance using the Shapiro–Wilk test and Levene’s test, respectively. For normally distributed data with homogeneous variance, differences among treatment groups were analyzed using one-way analysis of variance (ANOVA). When the overall treatment effect was significant, Tukey’s multiple comparison test was used for pairwise comparisons. For data that did not meet parametric assumptions, the Kruskal–Wallis test followed by Dunn’s post hoc test was used.

To evaluate dose–response patterns across the equally spaced kaempferol concentrations of 0, 25, 50, 75, and 100 μg/mL, orthogonal polynomial contrasts were performed. The dose effect was partitioned into linear, quadratic, cubic, and quartic components using five-level orthogonal polynomial contrasts. The overall treatment effect was reported as P(dose), and the orthogonal polynomial contrasts were reported as P-linear, P-quadratic and P-cubic. Quadratic polynomial fitting was also performed to visualize nonlinear dose–response patterns, and the fitted curves were provided in Supplementary Figure S1.

For two-group comparisons between the control group and the selected 25 μg/mL kaempferol-treated group in subsequent acrosome integrity, proteomic, and metabolomic analyses, an independent-samples *t*-test was used for normally distributed data, whereas the Mann–Whitney U test was used for non-normally distributed data. Statistical significance was set at *p* < 0.05. Statistical analyses were performed using R software 4.6.0 and GraphPad Prism 8.0.

## 3. Results

### 3.1. Effects of Different Concentrations of Kaempferol on Motility Parameters of Frozen-Thawed Semen from Dairy Rams

As shown in Table 1, kaempferol supplementation significantly affected several motility parameters of frozen-thawed semen from Yuansheng Aite dairy rams. Among the measured parameters, PM, VCL, and VAP were significantly affected by kaempferol treatment (*p* = 0.014, *p* = 0.008, and *p* = 0.046, respectively). In contrast, RPM, LIN, WOB, and VSL were not significantly affected by treatment (*p* > 0.05).

Compared with the control group, the 25 μg/mL kaempferol group showed the highest observed mean values for PM, RPM, VCL, VAP, and VSL. However, increasing the concentration above 25 μg/mL did not consistently produce further improvement in sperm motility parameters. The 50, 75, and 100 μg/mL groups showed variable responses among different parameters, indicating that the motility response to kaempferol was not simply enhanced with increasing concentration.

Orthogonal polynomial analysis was further performed across the equally spaced kaempferol concentrations of 0, 25, 50, 75, and 100 μg/mL. No significant linear effect was observed for any motility parameter (P-linear > 0.05), suggesting that the response was not characterized by a simple dose-dependent increase or decrease. A significant quadratic effect was detected for VCL (P-quadratic = 0.012), indicating a nonlinear dose–response pattern for this parameter. In addition, PM, VCL, and VAP showed significant cubic components (P-cubic = 0.030, P-cubic = 0.024, and P-cubic = 0.045, respectively), further suggesting that the response of sperm motility parameters to kaempferol supplementation was complex and parameter-dependent.

Taken together, 25 μg/mL kaempferol showed the most favorable observed overall motility profile among the tested concentrations, although the polynomial analysis did not support defining it as an absolute optimal dose. Therefore, 25 μg/mL was selected as the representative treatment concentration for subsequent oxidative stress, energy metabolism, acrosome integrity, proteomic, and metabolomic analyses. The fitted orthogonal polynomial dose–response curves are provided in Supplementary Figure S1 for visualization.

### 3.2. Effects of Different Concentrations of Kaempferol on Oxidative Stress and Energy Metabolism in Frozen-Thawed Semen of Dairy Rams

**Figure 1:**
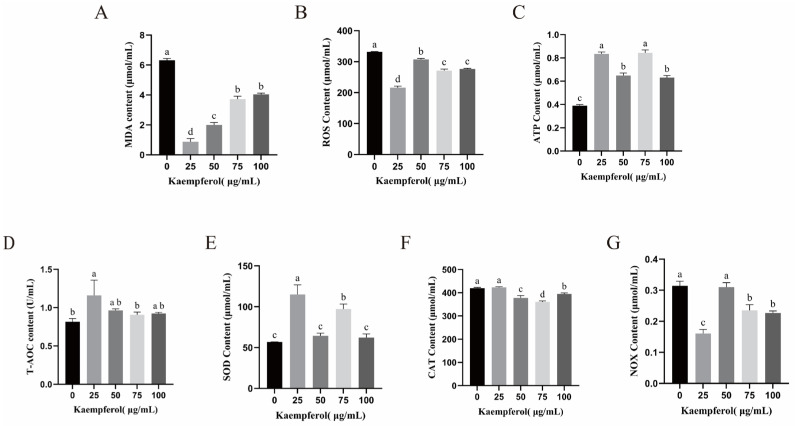
Effects of different kaempferol concentrations on oxidative stress and energy metabolism indicators in frozen-thawed semen from Yuansheng Aite dairy rams. (**A**), malondialdehyde (MDA) content; (**B**), reactive oxygen species (ROS) level; (**C**), adenosine triphosphate (ATP) content; (**D**), total antioxidant capacity (T-AOC); (**E**), superoxide dismutase (SOD) activity; (**F**), catalase (CAT) activity; (**G**), NADPH oxidase (NOX) level. Data are presented as mean ± SD. Different lowercase letters indicate significant differences among treatment groups (*p* < 0.05).

The effects of different kaempferol concentrations on oxidative stress and energy metabolism indicators in frozen-thawed semen from dairy rams are shown in Figure 1. Compared with the control group, kaempferol supplementation generally reduced oxidative stress-related indicators, including MDA, ROS, and NOX, and increased antioxidant-related indicators, including T-AOC and SOD activity. Among the tested concentrations, 25 μg/mL showed the most favorable overall antioxidant profile, as reflected by lower oxidative stress marker levels and higher antioxidant capacity.

However, the responses of oxidative stress and energy metabolism indicators were not uniformly enhanced with increasing kaempferol concentration. Higher concentrations did not consistently produce stronger protective effects than 25 μg/mL. CAT activity showed no obvious difference among treatment groups. Regarding energy metabolism, ATP content was increased in kaempferol-treated groups compared with the control group, but the response varied among concentrations.

Taken together, the 25 μg/mL group showed the most favorable overall pattern in terms of reduced oxidative stress, enhanced antioxidant activity, and improved energy metabolism. Therefore, this concentration was selected as the representative kaempferol treatment group for subsequent mechanistic analyses.

### 3.3. Effects of 25 μg/mL Kaempferol on the Metabolomic Profile of Frozen-Thawed Semen

Based on the results of sperm motility, oxidative stress, and energy metabolism analyses, 25 μg/mL kaempferol was selected as the representative treatment concentration for subsequent metabolomic analysis. Untargeted metabolomic profiling was performed to compare frozen-thawed semen samples from the control group (CK) and the 25 μg/mL (TG) kaempferol-treated group.

**Figure 2:**
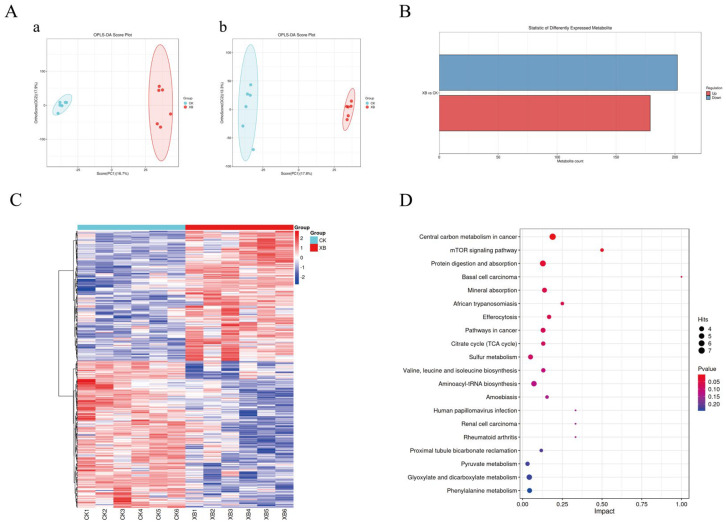
Metabolomic profiling of frozen-thawed semen from Yuansheng Aite dairy rams after 25 μg/mL kaempferol treatment. (**A**), OPLS-DA score plot of the control group and the 25 μg/mL kaempferol-treated group, (**a**) represents the positive ion mode, and (**b**) represents the negative ion mode; (**B**), numbers of upregulated and downregulated differential metabolites; (**C**), hierarchical clustering heatmap of differential metabolites; (**D**), KEGG pathway enrichment analysis of differential metabolites. CK, control group; TG, 25 μg/mL kaempferol-treated group. Differential metabolites were identified using VIP > 1 and *p* < 0.05.

OPLS-DA showed a clear separation between the control and kaempferol-treated groups, indicating that 25 μg/mL kaempferol was associated with marked changes in the metabolic profile of frozen-thawed semen. The permutation test indicated that the model was not overfitted, with R^2^Y = 0.982 and Q^2^Y = 0.926 (Figure 2A). Based on VIP > 1 and *p* < 0.05, a total of 381 differential metabolites were identified in the kaempferol-treated group compared with the control group, including 179 upregulated and 202 downregulated metabolites (Figure 2B).

Hierarchical clustering analysis showed distinct metabolic patterns between the control and kaempferol-treated groups, with good consistency among biological replicates within each group (Figure 2C). KEGG enrichment analysis showed that the differential metabolites were mainly enriched in pathways related to central carbon metabolism, glycolysis/gluconeogenesis, the pentose phosphate pathway, amino acid metabolism, lipid metabolism, and mineral absorption. Among these pathways, central carbon metabolism showed the highest pathway impact and enrichment significance (*p* < 0.01; Figure 2D). These results suggest that 25 μg/mL kaempferol was associated with metabolic remodeling in frozen-thawed dairy ram semen.

### 3.4. Effects of 25 μg/mL Kaempferol on the Proteomic Profile of Frozen-Thawed Semen

Proteomic analysis was performed to investigate protein-level changes associated with 25 μg/mL kaempferol treatment in frozen-thawed semen from Yuansheng Aite dairy rams. Frozen-thawed semen samples from the control group (CK) and the 25 μg/mL kaempferol-treated group (TG) were compared. Differentially expressed proteins were identified using the criteria of fold change ≥ 1.5 or ≤0.667 and *p* < 0.05.

**Figure 3:**
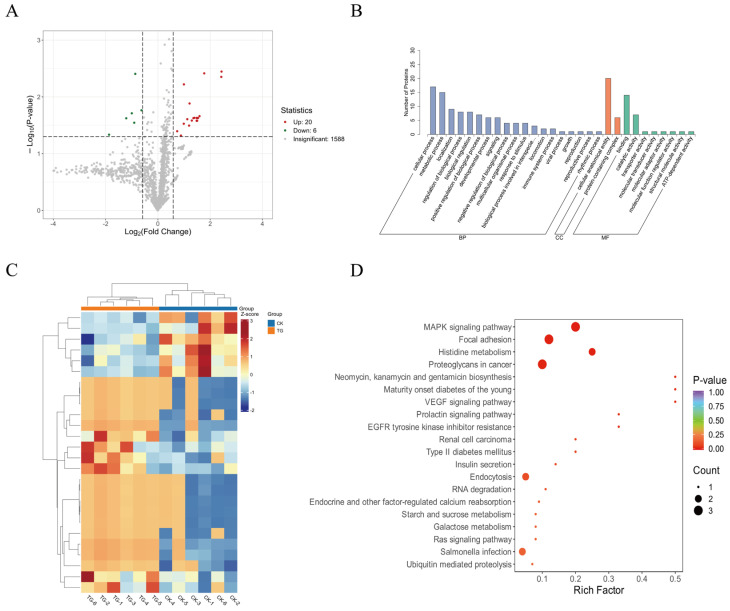
Proteomic profiling of frozen-thawed semen from Yuansheng Aite dairy rams after 25 μg/mL kaempferol treatment. (**A**), volcano plot of differentially expressed proteins between the control group and the 25 μg/mL kaempferol-treated group; (**B**), hierarchical clustering heatmap of differentially expressed proteins; (**C**), GO functional enrichment analysis of differentially expressed proteins; (**D**), KEGG pathway enrichment analysis of differentially expressed proteins. CK, control group; TG, 25 μg/mL kaempferol-treated group. Differentially expressed proteins were identified using fold change ≥ 1.5 or ≤0.667 and *p* < 0.05.

As shown in Figure 3A, volcano plot analysis identified 26 differentially expressed proteins between the control and kaempferol-treated groups, including 20 upregulated and 6 downregulated proteins. Hierarchical clustering analysis showed distinct protein expression patterns between the two groups, with good consistency among biological replicates within each group (Figure 3B).

GO functional enrichment analysis showed that the differentially expressed proteins were mainly associated with sperm cytoskeletal organization, redox homeostasis, acrosomal structure, and sperm motility-related functions (Figure 3C). KEGG pathway enrichment analysis indicated that these proteins were enriched in pathways related to focal adhesion, MAPK signaling, actin cytoskeleton regulation, and oxidative phosphorylation (Figure 3D). These results suggest that 25 μg/mL kaempferol treatment was associated with changes in proteins related to sperm structure, energy metabolism, and stress response after cryopreservation.

**Figure 4:**
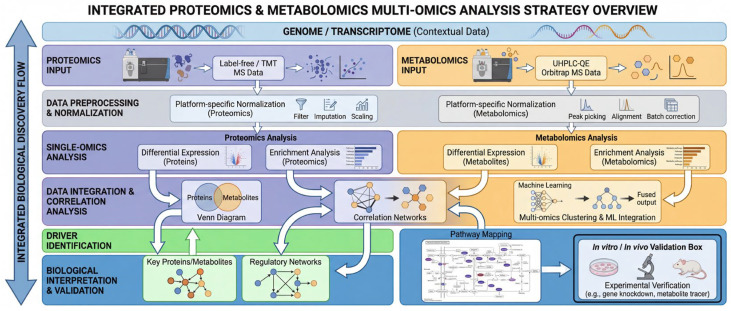
Workflow of the integrated proteomic and metabolomic analysis used to investigate potential mechanisms associated with kaempferol-mediated protection in frozen-thawed semen from Yuansheng Aite dairy rams.

To further integrate the metabolomic and proteomic results, an integrated multi-omics analysis workflow was applied. The workflow included data preprocessing, differential molecule screening, pathway enrichment analysis, protein-metabolite association analysis, and biological interpretation related to sperm cryotolerance (Figure 4).

### 3.5. Effect of 25 μg/mL Kaempferol on Acrosome Integrity of Frozen-Thawed Semen

Based on the proteomic and metabolomic results, acrosome integrity was further evaluated to assess whether 25 μg/mL kaempferol was associated with improved structural preservation of frozen-thawed sperm. FITC-PNA/DAPI dual-fluorescence staining was used to compare sperm acrosome integrity between the control group and the 25 μg/mL kaempferol-treated group.

**Figure 5:**
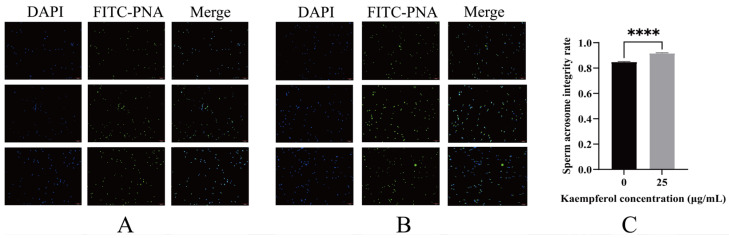
Effect of 25 μg/mL kaempferol on acrosome integrity in frozen-thawed semen from Yuansheng Aite dairy rams. (**A**), representative FITC-PNA/DAPI fluorescence images of spermatozoa from the control group; (**B**), representative FITC-PNA/DAPI fluorescence images of spermatozoa from the 25 μg/mL kaempferol-treated group; (**C**), quantitative analysis of acrosome integrity. Blue fluorescence indicates sperm nuclei stained with DAPI, and green fluorescence indicates acrosomal staining with FITC-PNA. **** *p* < 0.001.

As shown in Figure 5A,B, spermatozoa in the control group showed more obvious acrosomal damage, including incomplete acrosomal fluorescence, fluorescence diffusion, and disrupted acrosomal boundaries. In contrast, spermatozoa treated with 25 μg/mL kaempferol showed clearer and more continuous green fluorescence in the acrosomal region.

Quantitative analysis showed that acrosome integrity was significantly higher in the 25 μg/mL kaempferol-treated group than in the control group (*p* < 0.001; Figure 5C). These results indicate that 25 μg/mL kaempferol was associated with improved acrosomal structural preservation in frozen-thawed semen from Yuansheng Aite dairy rams.

## 4. Discussion

Sperm cryopreservation constitutes a core technology for the spatio-temporal conservation and efficient utilization of superior livestock germplasm resources [13,45]. However, multiple insults induced during the freezing-thawing process, including oxidative stress, cytoskeletal damage, membrane destabilization, and ATP-related energy disturbance, significantly impair post-thaw sperm motility, membrane integrity, and fertilization capacity [46]. This represents a critical bottleneck constraining the broader application of ram semen cryopreservation [46,47,48]. Under cryopreservation stress, the dynamic equilibrium between oxidative and antioxidant systems within spermatozoa is disrupted. Excessive ROS accumulation induces membrane lipid peroxidation and subcellular structural damage [31]. Concurrently, cryopreservation-related oxidative stress may impair mitochondrial-associated energy metabolism and further aggravate sperm functional decline [16,31]. Mechanical stress and osmotic pressure fluctuations during freezing and thawing can also compromise the sperm cytoskeleton and acrosome structure, thereby reducing cryotolerance and physiological function. These findings are consistent with recent studies emphasizing the multifactorial nature of sperm cryoinjury, in which oxidative damage, energy metabolic disturbance, and structural destabilization are closely interconnected mechanisms driving poor post-thaw quality [49,50].

This study, using the domestically bred Yuansheng Aite dairy sheep as the experimental model, systematically evaluated the effects of different kaempferol concentrations on semen cryopreservation quality. According to the revised statistical analysis, kaempferol treatment significantly affected PM, VCL, and VAP, whereas RPM, LIN, WOB, and VSL were not significantly affected. Orthogonal polynomial analysis further showed no significant linear effect for any motility parameter, while VCL exhibited a significant quadratic response. Therefore, the present results do not support defining 25 μg/mL as an absolute mathematically optimal dose. Instead, 25 μg/mL should be interpreted as the concentration showing the most favorable observed overall motility profile under the present experimental conditions and was therefore selected as the representative concentration for subsequent oxidative stress, ATP-related energy metabolism, acrosome integrity, proteomic, and metabolomic analyses.

This distinction is important because sperm quality after cryopreservation is a multifactorial outcome and cannot be fully explained by a single motility parameter or a single fitted dose–response curve. Although 25 μg/mL showed the highest observed mean values for several motility parameters, the polynomial analysis indicated that the response pattern was complex and parameter-dependent. Similar interpretations have been adopted in recent semen cryopreservation studies, where moderate concentrations of cryoprotective or antioxidant additives produced the most consistent overall improvements, even when not all parameters responded significantly or linearly. Such a nonlinear and parameter-dependent response is also biologically plausible because kaempferol may exhibit antioxidant or pro-oxidant effects depending on its concentration and the surrounding redox conditions [51]. For example, a recent study on MitoQ in cryopreserved canine sperm reported concentration-dependent effects, with a moderate concentration producing the most consistent improvement in post-thaw quality, whereas the highest concentration was associated with impaired membrane integrity and increased oxidative stress markers [52]. Similarly, in rooster sperm cryopreservation, DMA affected motility, viability, antioxidant biomarkers, and gene expression through linear and/or quadratic trends, indicating that sperm responses to extender additives may vary across endpoints rather than follow a simple dose-increasing pattern [53].

The favorable performance observed at 25 μg/mL may be related to the ability of kaempferol to restore redox balance during freezing and thawing. As a flavonoid antioxidant, kaempferol may scavenge excessive ROS, reduce lipid peroxidation, and reinforce endogenous antioxidant defense, thereby contributing to better preservation of sperm motility and structural integrity [32,51]. However, spermatozoa require a finely regulated redox environment for normal physiological signaling. Therefore, excessive antioxidant supplementation may not provide additional protection and may even disturb redox homeostasis, interfere with ROS-mediated sperm signaling, or reduce the adaptive oxidative balance required for normal sperm function. This may explain why higher kaempferol concentrations did not consistently improve motility parameters beyond the 25 μg/mL group. The present findings are also consistent with the concept of a narrow effective concentration range for antioxidant supplementation in semen cryopreservation. Accordingly, the biological relevance of 25 μg/mL kaempferol lies not in being a universally optimal dose, but in producing the most favorable observed balance among motility performance, oxidative stress attenuation, antioxidant defense, ATP-related energy status, and acrosomal preservation in this study.

Notably, CAT activity in kaempferol-treated groups showed no significant difference from the control group, suggesting that kaempferol-mediated antioxidant regulation in frozen-thawed ram semen may mainly involve ROS scavenging and reinforcement of selected endogenous antioxidant components, particularly SOD-related superoxide detoxification, rather than broad activation of all antioxidant enzymes. Similar selective antioxidant responses have also been observed in recent ram semen cryopreservation studies using other natural antioxidants [54]. This result further supports the view that antioxidant supplementation does not necessarily produce uniform changes in all redox-related markers. In addition, sperm motility is influenced not only by ATP content but also by membrane stability, flagellar cytoskeletal integrity, and acrosome structural integrity [55,56,57]. Therefore, the improvement in sperm motility observed in the 25 μg/mL group likely reflected coordinated preservation of redox balance, membrane functionality, ATP-related energy status, and flagellar structural integrity rather than a single biochemical change. This interpretation is consistent with current sperm cryobiology, in which post-thaw motility is considered a multifactorial trait rather than an isolated energetic endpoint [49].

To further elucidate the potential protective mechanism of kaempferol, this study conducted combined proteomic and metabolomic analyses on frozen-thawed semen from the 25 μg/mL kaempferol-treated group and the control group. Proteomic analysis showed that 25 μg/mL kaempferol treatment altered the protein expression profile of frozen-thawed dairy ram semen. The 26 differentially expressed proteins identified were enriched in pathways including focal adhesion, actin cytoskeleton regulation, and oxidative phosphorylation-related energy metabolism. Pathways related to focal adhesion and actin cytoskeleton organization provide a biologically coherent link between oxidative injury and structural deterioration of spermatozoa during cryopreservation. Preservation of these pathways may contribute to the maintenance of acrosomal stability, flagellar organization, and post-thaw motility [58]. Previous studies suggest that focal-adhesion-associated signaling participates in sperm actin remodeling and acrosome-related events; however, its direct role in kaempferol-mediated protection during ram sperm cryopreservation remains to be experimentally validated. Dysfunction of focal adhesion kinase (FAK) can affect downstream signaling related to actin polymerization, sperm tail cytoskeleton integrity, acrosome structure, and sperm motility [59]. Although direct evidence in cryopreserved ram sperm remains limited, recent studies increasingly support the importance of cytoskeleton-associated regulation in maintaining sperm functional competence. Accordingly, the enrichment of focal adhesion-related proteins in the present study should be interpreted as mechanistically informative but still hypothesis-generating rather than conclusive proof of direct target regulation by kaempferol [60].

Excessive ROS generated during cryopreservation can induce oxidative modification of sperm proteins and may affect signaling proteins involved in cell–matrix or cytoskeleton-related pathways [61,62,63]. Combined with the antioxidant indicator results in the present study, we hypothesize that kaempferol may reduce oxidative stress during freezing and thawing, thereby helping to preserve proteins associated with focal adhesion and actin cytoskeletal remodeling. This may contribute to the maintenance of sperm acrosome and flagellar structure and alleviate the combined damage caused by ice crystal mechanical stress and oxidative stress. However, the specific molecular regulatory axis and core targets require subsequent functional validation. Therefore, the proteomic findings should be interpreted as pathway-level associations that generate mechanistic hypotheses rather than as definitive evidence of causal regulation.

Metabolomic analysis indicated that kaempferol treatment was associated with remodeling of the metabolic profile of frozen-thawed ram sperm. Differential metabolites were enriched in pathways related to central carbon metabolism, glycolysis/gluconeogenesis, the pentose phosphate pathway, amino acid metabolism, lipid metabolism, and mineral absorption. Among these pathways, central carbon metabolism showed the highest enrichment significance. This metabolic pattern is important because glycolysis, oxidative phosphorylation, and the pentose phosphate pathway jointly contribute to sperm motility, redox balance, ATP generation, and functional competence [64,65,66]. In mammalian spermatozoa, ATP is mainly produced through glycolysis and mitochondrial oxidative phosphorylation, whereas cryopreservation-induced oxidative stress can disrupt energy homeostasis and impair sperm motility [64,65,66]. Excessive ROS may also oxidatively inactivate key enzymes in glycolysis and the pentose phosphate pathway, thereby altering carbohydrate metabolic flux and NADPH production [67,68,69]. Therefore, the simultaneous improvement in antioxidant markers and ATP content observed in the present study supports an association between kaempferol treatment and improved ATP-related energy metabolic status. However, these results do not establish direct mitochondrial protection. Targeted metabolite quantification, functional assays of glycolytic and pentose phosphate pathway enzymes, and mitochondrial-specific assays are still needed to validate this hypothesis.

In this study, 25 μg/mL kaempferol significantly enhanced acrosome integrity in frozen-thawed sperm. This finding provides cellular structural evidence consistent with the omics results. The acrosome is essential for the acrosome reaction and fertilization, and its integrity is closely associated with sperm fertilization potential [70]. The protective effect of kaempferol on acrosome integrity further supports its role in structural preservation during cryopreservation. This result is also consistent with antioxidant-based ram semen studies in which improved redox control during cryopreservation was accompanied by better preservation of membrane or acrosomal integrity [54]. Nevertheless, because fertilization rate and conception outcomes were not assessed in the present study, these findings should be interpreted as evidence of improved structural cryosurvival rather than direct proof of enhanced fertility.

This study has several limitations. First, the protective effect of kaempferol was evaluated mainly through in vitro sperm quality indicators, and its effects on fertilization potential, conception rate, and offspring outcomes were not verified through in vitro fertilization or in vivo artificial insemination trials. Second, the multi-omics analysis suggested potential associations involving focal adhesion, cytoskeletal organization, and central carbon metabolism, but the causal regulatory relationships and core molecular targets of kaempferol were not validated by pathway-specific inhibitors, targeted metabolite quantification, protein functional assays, or genetic interventions. Third, mitochondrial membrane potential, mitochondrial ROS production, mitochondrial DNA integrity, and mitochondrial ultrastructure were not directly assessed; therefore, ATP content should be interpreted as an energy metabolism indicator rather than direct evidence of mitochondrial protection. In addition, because pooled ejaculates were used during the concentration-screening stage, individual differences in sperm freezability and antioxidant responsiveness may have been partially masked. Future studies should therefore confirm the reproducibility of the 25 μg/mL kaempferol response using individual ram ejaculates, additional intermediate concentrations around the apparent effective range, larger numbers of biological replicates, and fertility-oriented endpoints. These studies will be necessary to refine kaempferol supplementation strategies and to determine its practical reproductive value for Yuansheng Aite dairy ram semen cryopreservation.

## 5. Conclusions

In conclusion, kaempferol supplementation influenced the post-thaw quality of semen from Yuansheng Aite dairy rams, with 25 μg/mL showing the most favorable overall profile in sperm motility, oxidative stress status, ATP-related energy metabolism, and acrosome integrity under the present experimental conditions. Proteomic and metabolomic analyses further suggested that the protective effect of kaempferol may be associated with the regulation of pathways related to focal adhesion, cytoskeletal organization, oxidative phosphorylation, and central carbon metabolism. Therefore, moderate kaempferol supplementation may represent a potential antioxidant strategy for improving dairy ram semen cryopreservation. However, further studies using individual ram ejaculates and fertility-oriented endpoints are needed to confirm its practical reproductive benefits.

## Figures and Tables

**Table 1:** Effects of different kaempferol concentrations on motility parameters of frozen-thawed semen from Yuansheng Aite dairy rams.

Variable	0 μg/mL	25 μg/mL	50 μg/mL	75 μg/mL	100 μg/mL	P(dose)	P-linear	P-quadratic	P-cubic
PM	39.42 ± 0.62	58.67 ± 5.93	45.14 ± 9.52	51.66 ± 4.02	50.12 ± 1.45	0.014	0.173	0.093	0.030
RPM	17.49 ± 0.84	29.11 ± 3.51	22.40 ± 7.70	27.80 ± 7.70	22.04 ± 1.23	0.109	0.428	0.070	0.464
VCL	39.42 ± 0.62	70.02 ± 7.40	57.75 ± 13.40	61.00 ± 3.52	58.64 ± 6.79	0.008	0.062	0.012	0.024
VAP	20.10 ± 0.58	28.46 ± 1.42	23.59 ± 5.02	24.98 ± 2.58	24.60 ± 1.80	0.046	0.295	0.087	0.045
LIN	26.44 ± 3.82	26.02 ± 3.29	23.80 ± 0.28	26.14 ± 3.08	27.70 ± 3.56	0.654	0.649	0.229	0.858
WOB	41.59 ± 1.14	40.82 ± 2.64	39.06 ± 0.20	40.91 ± 2.47	42.19 ± 2.79	0.481	0.741	0.121	0.914
VSL	13.15 ± 1.29	18.07 ± 0.86	14.50 ± 3.13	15.98 ± 2.51	16.10 ± 1.36	0.107	0.326	0.321	0.082

Note: Values are presented as mean ± SD (*n* = 3). P(dose) indicates the overall treatment effect. P-linear, P-quadratic, and P-cubic indicate orthogonal polynomial contrasts across the equally spaced kaempferol concentrations. *p* < 0.05 was considered statistically significant. Sperm motility (PM, %), progressive motility (RPM, %), curved-line velocity (VCL, μm/s), aver-age path velocity (VAP, μm/s), linearity (LIN, %), wobble (WOB, %), and the following kinematic parameters: straight-line velocity (VSL, μm/s).

## Data Availability

The original contributions presented in this study are included in the article and Supplementary Materials. Further inquiries can be directed to the corresponding authors.

## Supplementary Materials

The following supporting information can be downloaded at: https://www.mdpi.com/article/10.3390/antiox15060773/s1, Figure S1. Orthogonal-polynomial dose response (frozen-thawed ram semen).

## Abbreviations

The following abbreviations are used in this manuscript:

mL	milliliter
μg	microgram
pH	potential of hydrogen
μm	Micrometre
*v*/*v*	Volume To Volume Ratio
mm	millimeter
i.d.	Inside Diameter
*m*/*z*	mass-to-charge ratio
log	logarithm
mM	millimolar
M	molar
μL	microliter
ng	nanogram
nm	nanometer
ROS	reactive oxygen species
PM	Sperm motility
RPM	Progressive motility
VSL	straight-line velocity
VCL	curved-line velocity
VAP	average path velocity
LIN	linearity
WOB	wobble
T-AOC	Total antioxidant capacity
MDA	Malondialdehyde
SOD	Superoxide dismutase
CAT	Catalase
NOX	NADPH oxidase
ATP	Adenosine triphosphate
LC-MS	Liquid Chromatograph Mass Spectrometer
MS	Mass Spectrometer
DEMs	Differentially expressed metabolites
VIP	variable importance in projection
*p*-value	Probability value
FDR	false discovery rate
SD	standard deviation
ANOVA	Analysis of Variance
CK	Control Check
TG	treatment group
OPLS-DA	Orthogonal partial least squares-discriminant analysis
DEPs	Difficult-to-express proteins
FAK	focal adhesion kinase
