# Supplementary material for: Effects of Kaempferol Supplementation on the Cryopreservation Quality of Semen from Yuansheng Aite Dairy Rams
Source: Antioxidants (Basel). 2026 Jun 22;15(6):773. doi: 10.3390/antiox15060773 (PMC13295986; doi:10.3390/antiox15060773)

Figure S1. Orthogonal−polynomial dose response (frozen−thawed ram semen)

Blue = fitted polynomial of the supported order; orange ... = predicted optimal dose

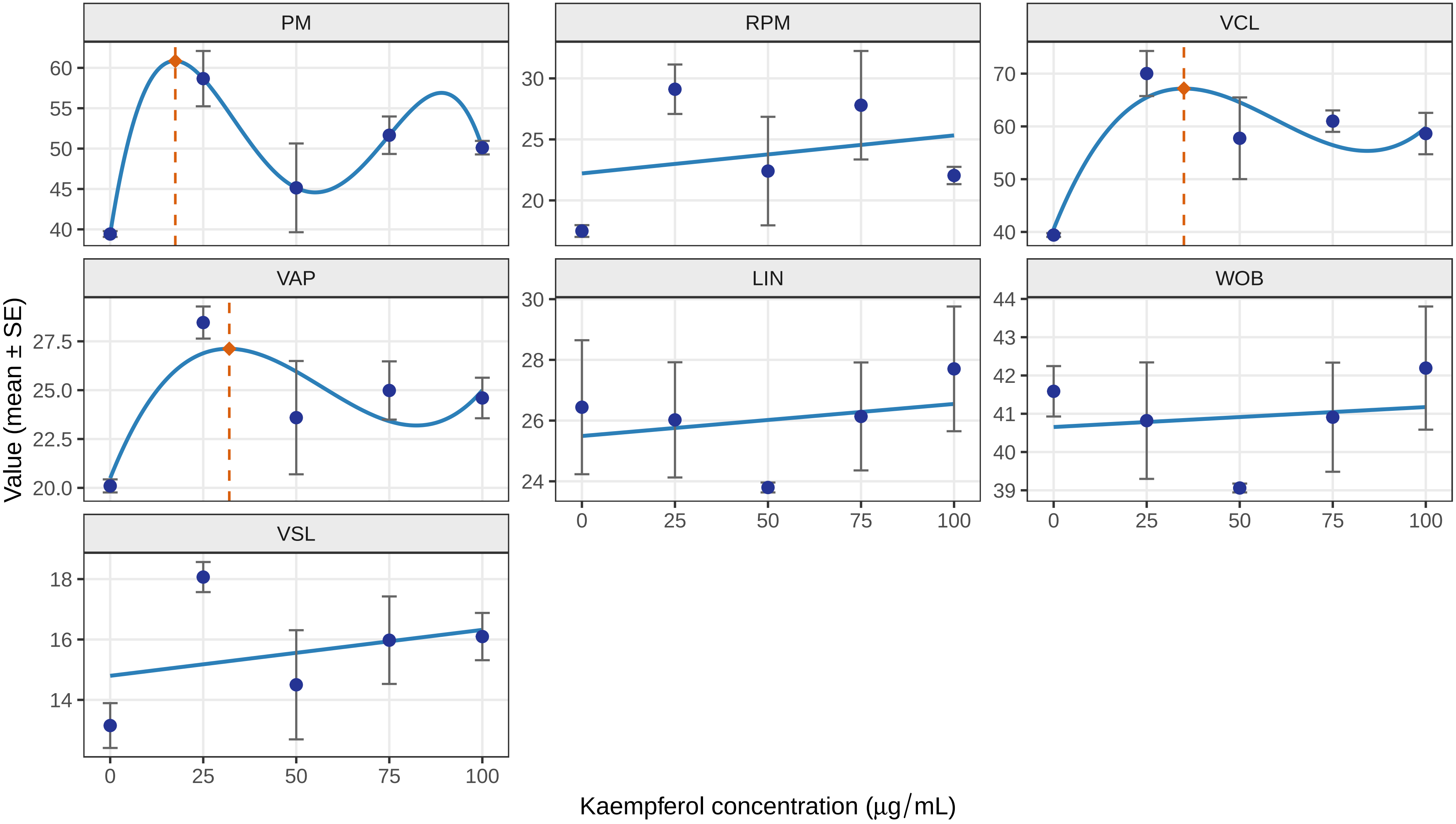

Supplement: Supplementary file 1 [file antioxidants-15-00773-s001.zip › antioxidants-4349082-supplementary.pdf]
